# Prolonged Deltamethrin Exposure Induces Dose-Dependent Glycerol Overproduction and Efficient Deltamethrin Removal by *Saccharomyces cerevisiae*

**DOI:** 10.3390/metabo16050305

**Published:** 2026-04-29

**Authors:** Mustafa Yavuz, Hakime Gül Yavuz, Recep Anil Kaya, Orhan Eren, Ceyhun Bereketoglu, Beste Turanli

**Affiliations:** 1 Department of Bioengineering, Faculty of Engineering, Marmara University, Istanbul 34854, Türkiye; mustafa-yavuz@tarimorman.gov.tr (M.Y.); recep.anil@gmail.com (R.A.K.); ceyhun.bereketoglu@marmara.edu.tr (C.B.); 2 Denizli Food Control Laboratory Directorate, Ministry of Agriculture and Forestry, Denizli 20010, Türkiye; hakimegul.yavuz@tarimorman.gov.tr; 3 TAGEM-Central Research Institute of Food and Feed Control, Ministry of Agriculture and Forestry, Bursa 16160, Türkiye; orhan.eren@tarimorman.gov.tr; 4 Health Biotechnology Common Application and Research Center of Excellence (SABIOTEK), Istanbul 34220, Türkiye

**Keywords:** Deltamethrin, *Saccharomyces cerevisiae*, fermentation, glycerol, glycerol 3-phosphate dehydrogenase, GPD1, GPD2

## Abstract

**Background/Objectives**: Pest management strategies rely on insecticides such as deltamethrin (DM), a commonly applied type II pyrethroid. As a natural component of food-associated microflora, *Saccharomyces cerevisiae* inevitably encounters DM residues in crops used for fermentation processes, including dough leavening and winemaking. However, the prolonged effect of DM exposure on yeast fermentation performance and its capacity to remove DM remained unclear. **Methods**: In this study, *S. cerevisiae* was continuously exposed to a non-lethal concentration (10 mg/L) and a low-inhibition toxic concentration (30 mg/L) of DM for 30 days. **Results**: Yeast exhibited high removal capacity, removing 98.05 ± 1.2% and 98.28 ± 0.4% of DM at 10 mg/L and 30 mg/L, respectively. Prolonged exposure to DM at both concentrations did not significantly affect biomass formation, glucose consumption, ethanol production, or acetic acid levels. In contrast, glycerol production increased markedly, reaching 1.1 g/L and 1.5 g/L in cultures exposed to 10 mg/L and 30 mg/L DM, respectively. Consistent with these changes, the expression levels of GPD1 and GPD2, which encode rate-limiting enzymes in glycerol biosynthesis, were upregulated in a dose-dependent manner. **Conclusions**: Given the fact that *Saccharomyces cerevisiae* is a workhorse for the biotechnological industry and has a wide range of applications, including in the food industry, elevated glycerol production in yeast under DM exposure is noteworthy in terms of yeast-based applications.

## 1. Introduction

**Figure 1:**
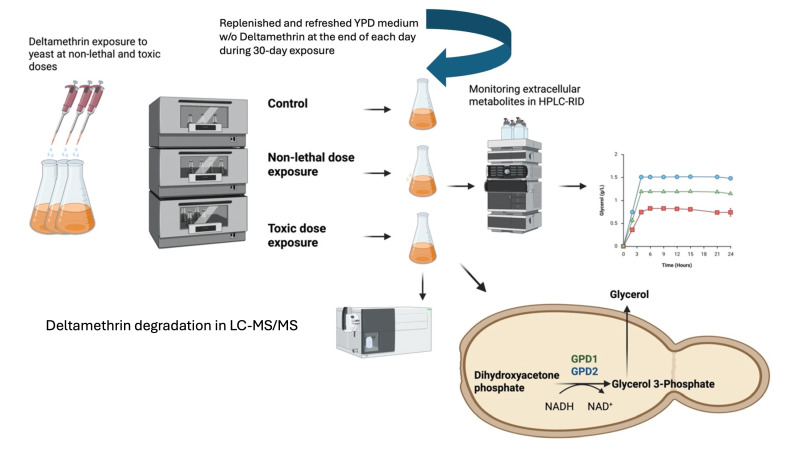
Schematic representation of dose-dependent response to DM exposure in yeast.

With the increasing global demand for food production, ensuring crop safety during the preharvest period and maintaining crop efficiency during the postharvest stage have become paramount [1]. In accordance with this, the integration of effective pest management strategies, including the application of pyrethroid insecticides, has been widely implemented. Pyrethroids are classified into two categories: Type I and Type II [2]. The distinction between the two types of pyrethroids is based on their chemical structures and mode of action on insects. Type II pyrethroids contain an alpha-cyano-3-phenoxybenzyl moiety whereas Type I pyrethroids do not have such a moiety [3,4]. Deltamethrin ([cyano-[3-(phenoxy)phenyl] methyl] 3-(2,2-dibromoethenyl)-2,2-dimethylcyclopropane-1-carboxylate) is classified as a Type II pyrethroid, which has not only been used in agricultural applications but also in aquaculture to eliminate arthropods that carry vector-borne parasitic diseases [5]. Despite its effectiveness against target organisms, the persistence of DM residues after treatment poses potential risks to human health and biodiversity due to off-target effects on non-target organisms [1,6]. *Saccharomyces cerevisiae* can be a non-target organism for DM exposure due to being naturally present in food microflora [7,8]. *S. cerevisiae* is extensively employed in industrial fermentation processes, particularly in bread and wine production. Therefore, understanding how pesticide residues affect yeast physiology and fermentation activity has become increasingly important. To that end, previous studies have investigated the impact of pesticide residues on yeast fermentation [7,8,9,10]. Six pesticides, including DM, were investigated during breadmaking and it was found that DM concentrations between 1 mg/kg and 4 mg/kg did not adversely affect yeast growth [9]. Similarly, no changes were observed in the fermentation performance of steamed bread prepared with flour derived from wheat grown on a farm treated with 25 g/L DM. Also, it was further demonstrated that fermentation of grapes containing 0.19 mg/kg DM did not impair yeast cellular activity [5]. While these studies suggest that DM has minimal effects on *S. cerevisiae* at relatively low concentrations, it was reported that baker’s yeast becomes susceptible to DM at higher concentrations. Following 2 h DM exposure, yeast mortality reached 9% and 12% for 20 mg/L and 40 mg/L DM, respectively [11]. Since *S. cerevisiae* serves as a well-established eukaryotic model organism for investigating xenobiotic modes of action, an exposure model in yeast for bisphenol-A was employed. In that study, the low-inhibition toxic concentration of bisphenol-A was defined as the exposure concentration through which cellular mortality remained below 10% of overall cells exposed to bisphenol-A, while the non-lethal concentration of bisphenol-A was adjusted to the highest applied bisphenol-A dose that did not cause any cellular death [12]. Based on this approach and the findings of [11], this study defined 30 mg/L DM as a low-inhibition toxic concentration and 10 mg/L as the highest non-lethal exposure concentration for yeast. Since it was reported that DM can remain in crops for up to 32 days [13], in this study, yeast strains that were continuously exposed to low-inhibition toxic and non-lethal DM concentrations for 30 days were assessed for their ability to remove DM from the fermentation medium. To ensure continuous exposure, fresh medium w/o DM was provided at the end of 24 h during 30 days. Subsequently, fermentation experiments of the strains (not) exposed to DM for 30 days were conducted under the same exposure conditions to assess cellular growth, glucose consumption, and extracellular metabolite production, including ethanol as the main fermentation product and acetic acid and glycerol as byproducts of fermentation (Figure 1). The results revealed a marked increase in glycerol production, which was associated with dose-dependent upregulation of the glycerol-3-phosphate dehydrogenase genes GPD1 and GPD2. To the best of our knowledge, this is the first study to evaluate both DM removal ability and the fermentation performance of yeast strains continually exposed to DM for a 30-day period.

## 2. Materials and Methods

### 2.1. Strains, Culture Conditions, and DM Treatment

The *Saccharomyces cerevisiae* BY4742 strain (Matα; his3Δ1; leu2Δ0; lys2Δ0; ura3Δ0), sourced from the Euroscarf collection (Frankfurt, Germany), was used as the model organism. A preculture was prepared by inoculating cells from a 30% glycerol stock stored at −80 °C. At 180 rpm and 30 °C, yeast was grown in Yeast Peptone Dextrose (YPD) medium (10 g/L yeast extract, 20 g/L peptone, 20 g/L glucose; Sigma-Aldrich, St. Louis, MO, USA). For the 30-day exposure, yeast cells were treated with either 10 mg/L or 30 mg/L deltamethrin (Sigma-Aldrich, St. Louis, MO, USA). Before starting the exposure experiments, we performed a fermentation in medium alone without yeast with 10 mg and 30 mg DM to ensure that self-degradation/removal did not occur due to the pure fermentation conditions (Figure S1). Every 24 h during the 30-day DM exposure, at the end of each day, 1% of the preculture was collected by centrifugation at 10,000 rpm for 10 min, washed twice with sterile YPD, and then inoculated into 25 mL of fresh YPD medium in a 125 mL flask with a corresponding replenished DM concentration. This was repeated during the 30-day exposure. Before the inoculation for the next loop, cellular growth was measured as OD_600nm_ (Figure S2). The yeast fermentation control group was only replenished with fresh YPD without DM. After 30 days of the non-DM treatment, yeast cells were grown in 2% YPD containing 10 mg/L, 30 mg/L, or no DM. Fermentation was initiated for 24 h when OD_600nm_ was 1. All experiments were performed in triplicate.

### 2.2. LC-MS/MS Analysis for DM Detection

For every treatment, DM was controlled in YPD medium. The extraction of DM from YPD medium was carried out according to a previous study [14]. An aliquot of the medium from the fermentation broth was diluted with MS-grade water (Merck, Darmstadt, Germany) to reach a working volume of 15 mL. The dilution was carried out based on the calibration points in LC-MS/MS analysis (0.1, 1, 5, 20, 40, 80, 100 ppb deltamethrin) with an acceptable linearity (r^2^ ≥ 0.99). Recovery rates were found within the range of 80–110%, and relative uncertainty was found at 20.01%. The diluted samples were shaken vigorously. An equal volume of extraction solvent, acetonitrile (Merck, Darmstadt, Germany) acidified with 1% glacial acetic acid (Merck, Darmstadt, Germany), was added to the mixture. The mixture of the extraction solvent and the sample was shaken for 1 min. A QuEChERS AOAC extraction kit (Agilent Technologies, Santa Clara, CA, USA) containing 6 g MgSO_4_ and 1.5 g sodium acetate was added to the mixture, then the tube was shaken for an additional 1 min. Following that, the centrifugation was done at 5000 rpm for 1 min; 8 mL was transferred to another tube containing 400 mg PSA, 400 mg C18, and 1200 mg MgSO_4_ to clean up the extract (Agilent Technologies, Santa Clara, CA, USA). The tube was shaken briefly and then centrifuged at 5000 rpm for 1 min; 500 μL of the supernatant was filtered using a PVDF syringe filter (0.22 μm) before inoculation. The filtered extract was mixed with 475 μL 10.53 mM 99% pure ammonium formate (Fluka, Buchs, Switzerland) in water and 25 μL acetonitrile to increase ionization of deltamethrin. A Shimadzu UFLC XR coupled to a Shimadzu LCMS-8050 triple quadrupole mass spectrometer (Shimadzu Corporation, Kyoto, Japan) was used to determine deltamethrin concentrations in YPD medium. Chromatographic separation of DM was performed through a Fusion-RP HPLC column (50 mm × 2.0 mm, 2.5-μm particle size) coupled to a C18 4 × 3 mm guard column (Phenomenex, Torrance, CA, USA) and 40 °C column oven. The flow rate was set to 0.4 mL min^−1^, and 5 µL of samples was injected onto the column. Totals of 1 mM ammonium formate in water (A) and 1 mM ammonium formate in methanol (B) were utilized as the mobile phases. The gradient conditions were employed as follows: 0–2.5 min, 0–90% B; 2.5–4.0 min, 90–90% B; 4.0–4.1 min, 90–5% B; 4.1–6.0 min, 5.0–5.0% B. The analyses were carried out in ESI positive and negative ion mode using multiple reaction monitoring (MRM) for quantitative measurement. Mass spectrometry variables were as follows: nebulizing gas flow, 3.0 L min^−1^; drying gas flow, 15 L min^−1^; interface voltage, 3.5 kV; detector voltage, 1.68 kV; DL temperature, 250 °C; and heat block temperature, 400 °C. All experiments were performed in triplicate.

### 2.3. HPLC-RID Analysis for Extracellular Metabolite Detection

Extracellular metabolites, including ethanol as the main fermentation product of glucose assimilation, glucose as the carbon source for yeast fermentation, and glycerol and acetic acid as byproducts of glucose fermentation, were determined as described previously [15]. A high-performance liquid chromatography (HPLC) system (Shimadzu Corporation, Kyoto, Japan) with a Refractive Index Detector (RID) was utilized with a Rezex ROA-Organic acid H^+^ (8%) column (Phenomenex, Torrance, CA, USA) to quantify extracellular metabolite fermentation medium. The flow rate was set to 0.6 mL min^−1^. Oven temperature was maintained at 50 °C. The calibration points for all analytes in HPLC-RID were set from 0.1 g/L to 2 g/L with a good linearity (r^2^ ≥ 0.997). Even if there was a peak quantified below 0.1 g/L of chromatograms of yeast strains, the peak was considered as not detected since its quantity was below the limit of quantification. All experiments were performed in triplicate.

### 2.4. Quantitative Real-Time Reverse Transcription PCR Analysis

For quantitative real-time reverse transcription PCR (qRT-PCR), cDNA was synthesized from total RNA extracted using the QuantiTect Reverse Transcription Kit (Qiagen, Valencia, CA, USA), following the manufacturer’s instructions. The qRT-PCR analysis was carried out with the LightCycler FastStart DNA Master PLUS SYBR Green I Kit (Roche Molecular Biochemicals, Mannheim, Germany), in accordance with the supplier’s protocol. The primers employed are listed in Table 1. Each sample was run in biologically separate triplicates, and gene expression fold changes were calculated using the 2^−ΔΔCt^ method, as described in [16]. All experiments were performed in triplicate.

### 2.5. Bioinformatic Analysis

The raw RNA sequencing files used in this study were retrieved from the GEO database with the accession number PRJNA1393177. The experimental design of the RNA sequencing harbors the raw sequencing files of the 10 mg DM, 30 mg DM, and non-treated groups. Kallisto was utilized to map raw reads to the *Saccharomyces cerevisiae* reference genome in the NCBI database with the accession number GCA_003086655.1 [17]. Based on the count data obtained from bioinformatic analysis, enrichment analysis was conducted through R packages(Version 4.1.0) [18].

### 2.6. Statistical Analysis

The comparison of fold changes for GPD1 and GPD2 genes was conducted using biorender.com with one-way ANOVA (Dunnett’s post-test) followed by Tukey’s multiple-comparison test to compare multiple groups. *p* values < 0.05 of two genes were utilized to find out statistical differences. If the *p* value is lower than 0.05, it is marked with an asterisk of corresponding value (* *p* value < 0.05, ** *p* value < 0.01, *** *p* value < 0.001). Unless there are no asterisks between expression levels, differences between expression levels are not statistically significant. All expression levels were compared between each other during statistical analysis.

## 3. Results

The non-lethal dose and low-inhibition toxic dose of DM were set as 10 mg/L and 30 mg/L according to the concentrations tested in [11]. Both concentrations and YPD medium were replenished and refreshed every 24 h. In doing so, DM was continuously applied to yeast for 30 days. Throughout the 30-day exposure, the leftover DM concentrations were monitored in LC-MS/MS at the end of each day (Table 2). At the end of the 30-day DM exposure, the 24 h fermentation profile of yeast exposed to both concentrations generated through the chromatographic analysis in HPLC-RID. On the basis of the increase in glycerol production during 24 h fermentation, the expression levels of the rate-limiting enzymes GPD1 and GPD2 in yeast exposed to the highest non-lethal dose and low-inhibition toxic concentrations were compared to the yeast strain that was not exposed to DM. We found that the rate-limiting enzymes of glycerol production GPD1 and GPD2 were regulated depending on DM dose. The results of these experiments are presented below. For 10 mg/L (non-lethal) and 30 mg/L (low-inhibition toxic) DM concentrations, the remaining DM concentrations at the end of each day were detected in LC-MS/MS. The lowest remaining DM concentrations were achieved in the 21st (0.195 ± 0.146 mg/L) and 20th (0.517 ± 0.042 mg/L) loops for non-lethal and low-inhibition toxic doses.

**Figure 2:**
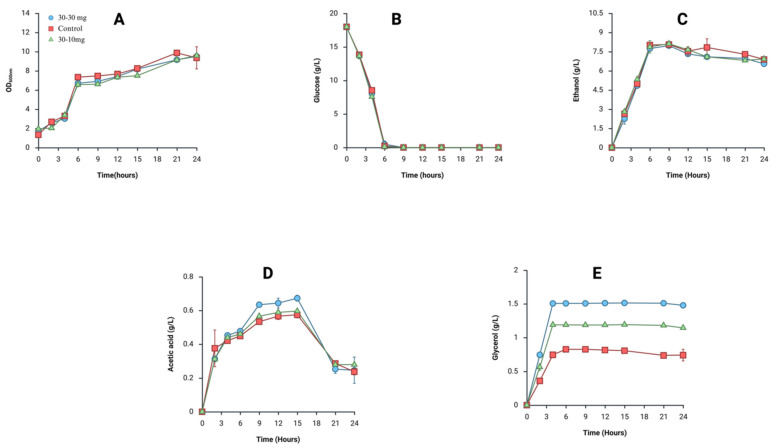
The fermentation profiles of yeast exposed to 10 mg/L and 30 mg/L DM. The cellular growth (**A**), glucose consumption (**B**), ethanol production (**C**), acetic acid production (**D**), and glycerol production (**E**) are plotted throughout the 24 h fermentation in YPD medium. 30–30 mg: yeast strain exposed to 30 mg/L DM for 30 days, 30–10 mg: yeast strain exposed to 10 mg/L DM for 30 days, control: yeast strain not exposed to DM.

To observe the effect of DM exposure on yeast fermentation, cellular growth, glucose consumption rate, and extracellular compounds produced during fermentation were profiled after chronic exposure to both doses for 30 days (Figure 2). There was no notable difference in terms of cellular growth (Figure 2A), glucose consumption (Figure 2B), and ethanol production (Figure 2C) among yeast strains tested in this study during the fermentation. Although the ethanol concentrations at the end of fermentation were similar in yeast strains, the ethanol concentrations at 15 h were found to be 7.15 ± 0.32 g/L for 30 mg/L DM-exposed yeast, 7.035 ± 0.21 g/L for 10 mg/L DM-exposed yeast, and 7.93 ± 0.48 g/L for yeast not exposed to DM (Figure 2D). There was, however, a dramatic increase in glycerol production in yeast exposed to DM regardless of the applied doses. The glycerol production by the yeast exposed to 30 mg/L DM reached 1.5 ± 0.01 g/L, and 1.1 ± 0.02 g/L glycerol was produced by the yeast exposed to 10 mg/L DM, while 0.8 ± 0.02 g/L glycerol was found in the fermentation medium of yeast not exposed to DM (Figure 2E).

**Figure 3:**
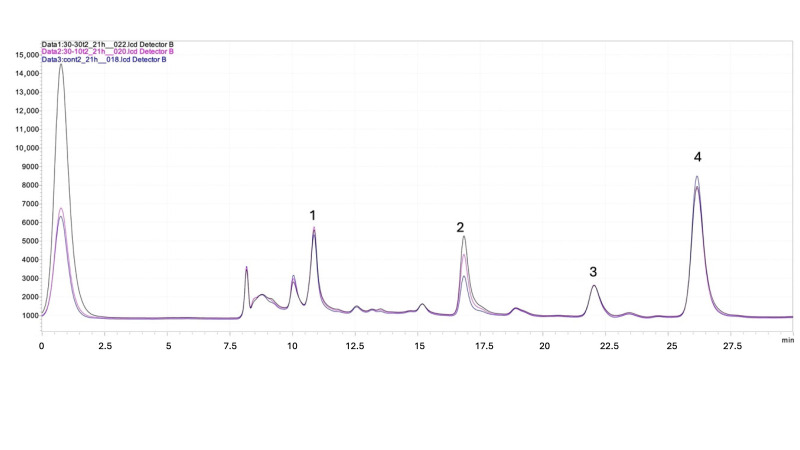
The overlapping chromatograms indicate the analytes detected in fermentation medium through HPLC-RID. For each peak, **1** denotes glucose, **2** denotes glycerol, **3** denotes acetic acid, and **4** denotes ethanol in 21 h fermentation data for 30 mg/L DM- and 10 mg/L DM-exposed yeast and non-exposed yeast. The chromatogram with a dark line (30_30t2_21h) represents the 30 mg/L DM-exposed yeast, the chromatogram with a pink line (30_10t2_21h) represents the 10 mg/L DM-exposed yeast, and the chromatogram with a blue line (cont2_21h) represents the non-exposed yeast.

The chromatographic overlapping of the yeast strains exposed to 30 mg/L and 10 mg/L DM and the control strain are shown in Figure 3.

**Figure 4:**
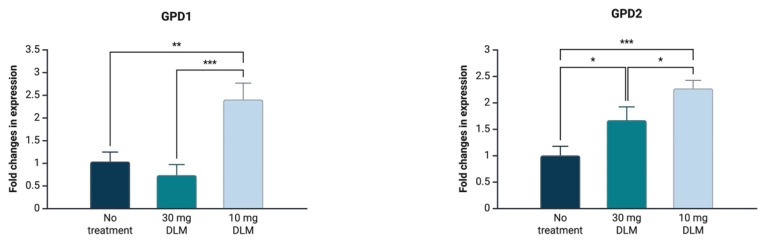
Quantitative real-time reverse transcription PCR analysis of two genes (GPD1, GPD2) related to glycerol production. The *p* value ranges, as a result of gene expression level comparisons, are statistically shown with asterisks (* *p* value < 0.05, ** *p* value < 0.01, *** *p* value < 0.001). Unless there are no asterisks between expression levels, differences between expression levels are not significant. Multiple comparisons were performed during statistical analysis.

Quantitative real-time reverse transcription PCR analysis of two genes (GPD1, GPD2) related to glycerol biosynthesis in yeast was employed to ascertain the elevated glycerol production in yeast exposed to DM. Regarding GPD1 expression levels, the 10 mg/L DM treatment led to overexpression, while there was no difference between the 30 mg/L DM treatment and the yeast that was not exposed to DM (Figure 4). On the contrary, GPD2 was upregulated in both yeast strains exposed to DM (Figure 4).

**Figure 5:**
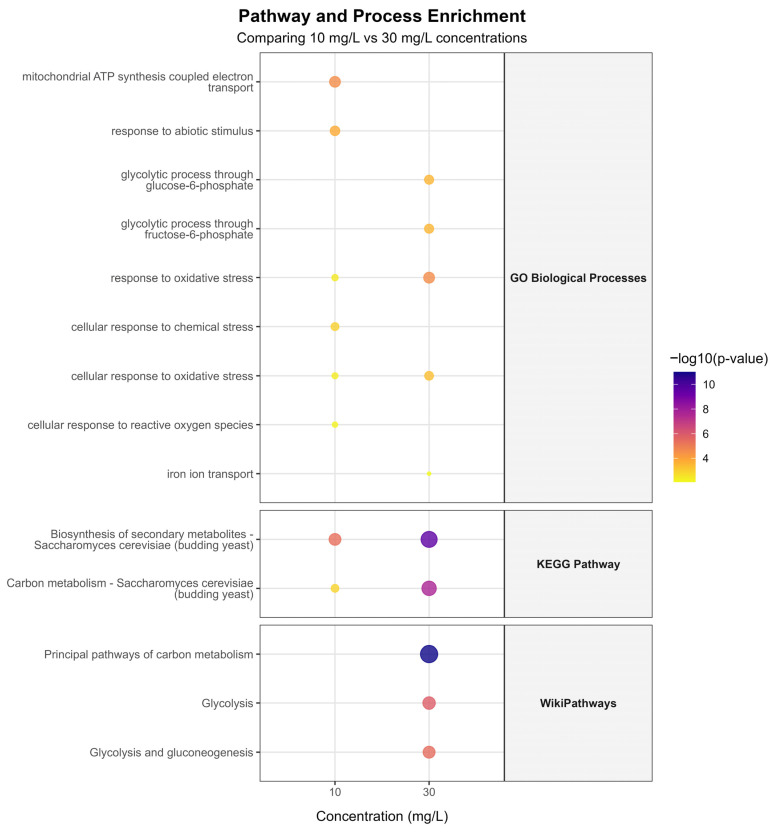
The enrichment analysis of raw RNA-seq data for yeast strains exposed to DM.

To elucidate the glycerol production of yeast, an enrichment analysis was carried out for the raw RNA-seq data for yeast strains (not) exposed to DM at both doses for 30 days that was obtained from the NCBI database with the accession number PRJNA1393177. The enrichment analysis indicated that 14 different pathways were influenced by DM exposure. RNA processing was found to be the most affected pathway, while xylose metabolism was the least affected metabolism among the 14 pathways (Figure 5).

## 4. Discussion

Owing to the health risks associated with pesticide residues in food and potential resistance development in insects, the biotransformation and biodegradation of DM has been a focal point of previous studies [7,8,9,17]. At 100 mg/L DM in the fermentation medium, *Lactiplantibacillus plantarum* was able to metabolize 86% of DM [17]. Given the high concentration applied in [19], an 89% DM (4 mg/kg) removal was reported in dough fermented with *S. cerevisiae* [9]. Also, 13-day fermentation with grapes naturally harboring *S. cerevisiae* led to complete DM (0.19 mg/kg) detoxification [7]. To evaluate whether *S. cerevisiae* subjected to 10 mg/L and 30 mg/L DM for 30 days removed DM, and to what extent, we monitored DM concentrations for 30 days via LC-MS/MS. Consistent with previous reports, the highest DM removal in this study was 98.05 ± 1.2% for the non-lethal dose, and 98.28 ± 0.4% for the low-inhibition toxic dose. Although a significant reduction in deltamethrin levels was observed in the presence of yeast during the 30-day exposure period, this study does not enable a clear differentiation between biodegradation and adsorption-based removal processes. The medium-only control groups showed that deltamethrin did not undergo abiotic degradation under the fermentation conditions, indicating that the decrease in deltamethrin levels is linked to the presence of Saccharomyces cerevisiae (Figure S1A). However, we cannot determine whether this removal is driven by metabolic change or by physical adsorption to yeast biomass from the present dataset. Determining the underlying mechanism would require further assays like the identification of potential degradation products, adsorption tests, or intracellular pesticide analyses. Consequently, while the findings indicate a substantial decrease in deltamethrin concentration during yeast fermentation, additional analyses are required to reveal the underlying mechanism behind this removal. To determine whether DM is completely removed and/or converted into its degradation products and how yeast enables this conversion and/or removal during DM exposure, further investigation is needed to determine potential DM degradation methods and products via yeast fermentation.

*Saccharomyces cerevisiae* is an industrially important organism, a workhorse for biotechnological applications including food fermentations and strain development for commodity production. In accordance with this, the capability of yeast assimilating carbon sources effectively to produce desirable products has been crucial for its industrial applications [15,19]. While previous reports indicated that DM exposure up to 4 mg/kg did not have any debilitating effects on fermentation capacity and efficiency [8], the effects of higher exposure levels on nutrient utilization and metabolite production has remained poorly understood. In accordance with previous reports, we found that biomass generation (Figure 2A), glucose consumption (Figure 2B), and acetic acid production rates (Figure 2C) were not influenced by DM exposure. Of note, there was a slight decrease in ethanol production at 15 h in yeast strains exposed to DM even though similar ethanol concentrations were obtained at the end of fermentation, possibly due to the ethanol uptake when glucose was depleted in the medium (Figure 2D). It is widely known that *S. cerevisiae* can uptake ethanol when available carbon sources are not present in the medium [20]. Since the ethanol production at the end of fermentation was similar in the yeast strains used in this study, it can be inferred that DM exposure might have delayed ethanol assimilation in yeast in the absence of available carbon sources. Also, the increase in glycerol production (Figure 2E) due to DM exposure shows that the metabolic flux could be diverted towards glycerol production instead of ethanol production. The strain that was not exposed to DM produced 0.8 g/L glycerol while yeast exposed to 10 mg/L and 30 mg/L DM produced 1.1 g/L and 1.5 g/L, respectively. All chromatograms of three strains overlapped in Figure 3, showing increased glycerol production in yeast exposed to DM regardless of the applied dose.

The GPD1 and GPD2 isogenes catalyze rate-limiting metabolic reactions for glycerol biosynthesis in yeast, converting glyceraldehyde-3-phosphate to glycerol with reducing NADH to NAD^+^. Even though there is a high similarity between these isogenes, their regulatory aspects significantly differ from each other [21,22]. It was reported that the upregulations of both enzymes were expressed depending on the stressors. If stressors act as osmatic stressors, GPD1 is upregulated. On the other hand, GPD2 was overexpressed in the absence of oxygen to maintain redox balance in yeast [23]. In this study, even though glycerol production (Figure 2E) increased in yeast exposed to DM regardless of DM dose, GPD1 and GPD2 expression levels were fine-tuned depending on the applied dose. Upon 10 mg DM exposure, GPD1 and GPD2 were upregulated; however, GPD1 upregulation was not observed in yeast exposed to 30 mg DM (Figure 4). The lack of GPD1 overexpression in yeast treated with 30 mg DM might be elucidated by the enrichment analysis shown in Figure 5. It is plausible that more metabolic flux towards glycerol might be produced under 30 mg DM exposure than 10 mg DM. Prior to generation of the substrate of glycerol biosynthesis, dihydroxyacetone phosphate, during glycolysis, glucose is converted to glucose-6-phoshate, fructose-6-phosphate, and fructose-1.6-biphospahte [24]. According to the enrichment analysis, 30 mg DM exposure had more impact on glycolysis metabolism in yeast than 10 mg exposure (Figure 5). The enrichment analysis indicated that glucose-6-phosphate and fructose-6-phosphate were affected by 30 mg DM exposure while no effect was found in 10 mg DM exposure. Fructose-6-phosphate is the precursor of fructose-1,6-biphosphate, and fructose-1,6-biphosphate is the precursor of dihydroxyacetone phosphate in glycerol metabolism. Therefore, it can be speculated that 30 mg DM exposure might lead to more dihydroxyacetone phosphate generation through the metabolic flux from glycolysis. Thus, yeast could manage excessive dihydroxyacetone phosphate through only GPD2 overexpression. Given the fact that GPD1 is mainly responsible for stress responses [25,26], and GPD2 is active in the presence of excessive redox imbalance [26], the glycerol overproduction in yeast exposed to 10 mg DM might occur due to stress, while the phenomenon during 30 mg DM exposure might be due to the increased metabolic flux in glycolysis. Also, it was shown that GPD1 cannot act as GPD2 under non-respiratory conditions, and GPD2 acts alone under non-respiratory conditions [25]. Corroborating this, 10 mg DM exposure affected mitochondrial ATP synthesis that is not functional under non-respiratory conditions (Figure 5). It might be inferred that 10 mg DM exposure led to aerobic fermentation while 30 mg DM exposure forced yeast into anaerobic fermentation through glycolysis. To elucidate the further distinction of GPD1 non-upregulation under 30 mg DM exposure, it might help us to seek the possible connection of which mechanism might provide non-respiratory conditions potentially created by 30 mg DM exposure.

It was found that rodents show increased GPD1 and GPD2 expression levels owing to DM exposure, corroborating the theory that DM exposure might cause an increase in glycerol production in yeast [27]. Accordingly, GPD1 upregulation in yeast led to up to 10-fold increased glycerol production, confirming that the increase in glycerol production arose from the activity of GPD1 [28]. Moreover, it was noted that GPD1 upregulation helped yeast resume growth in the face of myo-inositol synthase deletion to provide more dihydroxyacetone phosphate flux for glycerol production in yeast [28]. Yeast contributes to dough structure in the breadmaking process through its metabolites during fermentation. While CO_2_ and ethanol are the main metabolites shaping dough properties, acetic acid, succinic acid, and glycerol secondarily support dough to trap gas before baking. In the face of osmatic stress, yeast produces more glycerol during dough fermentation [28,29,30]. In accordance with this, GPD1 overexpression of yeast strains led to a 30% increase in glycerol production for bread dough fermentation [31], and a 10.66% increase in glycerol during wine fermentation [32]. At 30 mg/L DM exposure, the level of GPD1 expression in yeast was not statistically different from non-exposed yeast while GPD2 was upregulated (Figure 4B). These findings suggested that GPD1 may contribute to glycerol production under a low DM dose, whereas GPD2 may act in a more effective role for glycerol production in high-DM-dose exposure. Since GPD2 has been previously linked to maintaining intracellular NADH/NAD^+^ balance under stress conditions [24,25], the observed induction could suggest a shift in redox metabolism. However, this interpretation remains speculative and further analyses need to be performed to elucidate the potential correlation between DM exposure and redox imbalance in yeast.

Another aspect of glycerol overproduction in yeast is related to iron stress response [33,34]. It was reported that glycerol production in yeast was increased to 2 g/L from 1 g/L in the presence of iron as a stressor [34]. As a result of the enrichment analysis conducted in this study, carbon metabolism was highly regulated in the presence of DM regardless of the applied concentrations. Even though elevated glycerol production was previously associated with iron stress response [34], only 30 mg/L DM exposure altered iron transport regulation in yeast (Figure 5). The fact that iron stress due to DM exposure resulted in glycerol overproduction cannot be generalized, since the enrichment analysis of 10 mg DM did not provide any evidence regarding iron transport mechanisms. In line with this, the differences in glycerol production between the non-lethal dose and the low-inhibition toxic dose in this study might be due to the iron stress caused by DM toxicity.

## 5. Conclusions

In agricultural practices, pyrethroids have been applied to combat insects. A growing number of reports indicate that DM can also be present within non-target organisms [35]. *Saccharomyces cerevisiae*, found to facilitate fermentation in crop microflora, could be a non-target organism for DM treatment. In this study, yeast was exposed to DM at both a non-lethal dose and low-inhibition toxic dose for 30 days. We found that glycerol production was increased in yeast exposed to DM for 30 days, which was influenced by dose-dependent DM exposure. Taken together, DM exposure affected the fermentation capability of *Saccharomyces cerevisiae* by fine-tuning GPD1 and GPD2 in a dose-dependent manner. Even though glycerol production was high in these strains, there are reports indicating that the higher glycerol produced by yeast during fermentation could improve the textural and sensorial properties of bread and wine, respectively [28,31].

## Figures and Tables

**Table 1:** The list of primers used in this study for QPCR analysis of yeast samples.

Primes Name	Directions	Sequence
GPD1_Forward	5′-3′	CTGCCATCCAAAGAGTCGGT
GPD1_Reverse	5′-3′	GCGCAGGTGGTGATCAAATC
GPD2_Forward	5′-3′	TGGGATGGGGTAACAATGCC
GPD2_Reverse	5′-3′	GCAACACCAGCGGATTCTTG
ACT1_Forward	5′-3′	GCCTTCTACGTTTCCATCCA
ACT1_Reverse	5′-3′	GGCCAAATCGATTCTCAAAA

**Table 2:** The leftover DM concentrations of each loop after exposure at 10 mg/L and 30 mg/L doses.

Day	Remaining DM After 10 mg/L DM Exposure (mg/L)	Remaining DM After 30 mg/L DM Exposure (mg/L)
1	0.323 ± 0.162	1.314 ± 0.175
2	0.256 ± 0.209	1.458 ± 0.260
3	0.468 ± 0.003	1.314 ± 0.175
4	0.301 ± 0.028	1.458 ± 0.260
5	0.293 ± 0.111	2.015 ± 0.410
6	0.663 ± 0.161	2.616 ± 0.065
7	0.345 ± 0.039	1.806 ± 0.472
8	0.808 ± 0.148	0.989 ± 0.600
9	1.502 ± 0.160	1.389 ± 0.099
10	0.280 ± 0.027	1.772 ± 0.217
11	0.372 ± 0.202	1.897 ± 0.534
12	0.457 ± 0.419	1.681 ± 0.088
13	0.998 ± 0.290	1.706 ± 0.267
14	0.784 ± 0.148	1.295 ± 0.008
15	0.852 ± 0.211	0.824 ± 0.007
16	1.288 ± 0.368	1.063 ± 0.320
17	1.083 ± 0.061	1.337 ± 0.418
18	0.817 ± 0.196	0.909 ± 0.232
19	0.661 ± 0.018	0.651 ± 0.133
20	0.355 ± 0.127	0.517 ± 0.042
21	0.195 ± 0.160	0.435 ± 0.132
22	0.264 ± 0.007	0.454 ± 0.205
23	0.234 ± 0.148	0.437 ± 0.123
24	0.201 ± 0.028	0.455 ± 0.343
25	0.293 ± 0.111	0.456 ± 0.141
26	0.263 ± 0.161	0.486 ± 0.214
27	0.245 ± 0.039	0.472 ± 0.019
28	0.208 ± 0.148	0.485 ± 0.208
29	0.198 ± 0.146	0.486 ± 0.047
30	0.280 ± 0.027	0.443 ± 0.234

## Data Availability

Please add the corresponding content of this part.

## Supplementary Materials

The following supporting information can be downloaded at https://www.mdpi.com/article/10.3390/metabo16050305/s1. Figure S1 represents 0 h and 24 h data of the medium alone containing 10 mg and 30 mg deltamethrin, and shows deltamethrin peaks detected in LC-MS/MS. In the method, 523 m/z is considered the mother ion and 505 and 281 are set as daughter ions. Figure S2 demonstrates the cellular growth of yeast strains (not) exposed to DM for 30 days.

## Abbreviations

The following abbreviations are used in this manuscript:

DM	deltamethrin
GPD1	glycerol 3-phosphate dehydrogenase 1
GPD2	glycerol 3-phosphate dehydrogenase 2
