# Supplementary material for: Prolonged Deltamethrin Exposure Induces Dose-Dependent Glycerol Overproduction and Efficient Deltamethrin Removal by Saccharomyces cerevisiae
Source: Metabolites. 2026 Apr 29;16(5):305. doi: 10.3390/metabo16050305 (PMC13208566; doi:10.3390/metabo16050305)
Supplement: Supplementary file 1 [file metabolites-16-00305-s001.zip › metabolites-4240938-supplementary.pdf]

Prolonged Deltamethrin Exposure Induces Dose-Dependent Glycerol Overproduction and Efficient Deltamethrin Degradation by *Saccharomyces cerevisiae*

Mustafa Yavuz [1,2], Hakime Gül Yavuz [2], Recep Anil Kaya [1], Orhan Eren [3], Ceyhun Bereketoglu [1] and Beste Turanli*[1,4]

[1] Department of Bioengineering, Faculty of Engineering, Marmara University, Istanbul 34854, Türkiye

[2] Denizli Food Control Laboratory Directorate, Ministry of Agriculture and Forestry, Denizli 20010, Türkiye

[3] TAGEM-Central Research Institute of Food and Feed Control, Ministry of Agriculture and Forestry, Bursa 16160, Türkiye

[4] Health Biotechnology Common Application and Research Center of Excellence (SABIOTEK), Istanbul 34220, Türkiye

* Correspondence: beste.turanli@marmara.ed; Tel.: +90 216 777 3568

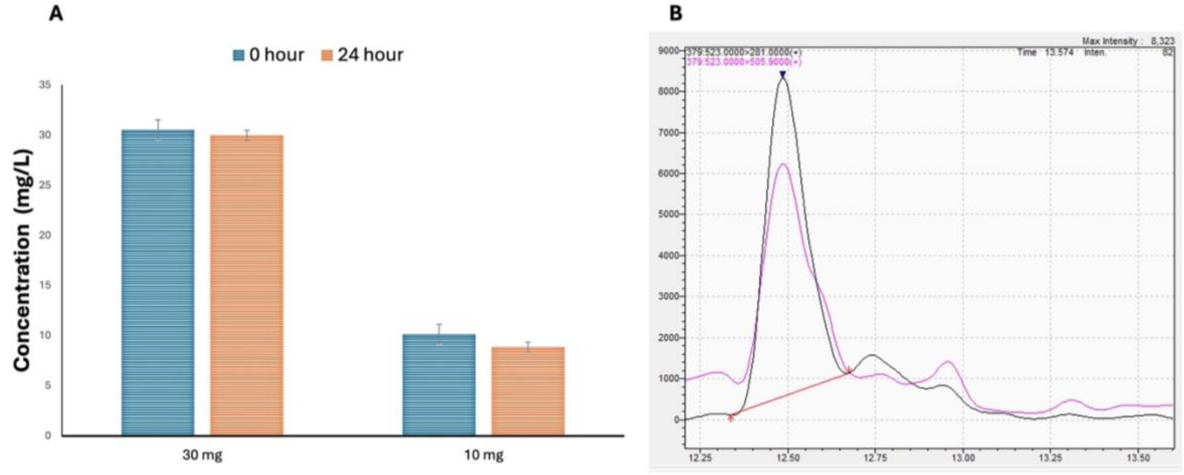

Figure S1: A indicates 0 hour and 24 hour data of the only-medium containing 10 mg and 30 mg Deltamethrin. B indicates Deltamethrin peaks detected in LC-MS/MS. 523 m/z is considered as mother ion and 505 and 281 are set as daughter ions.

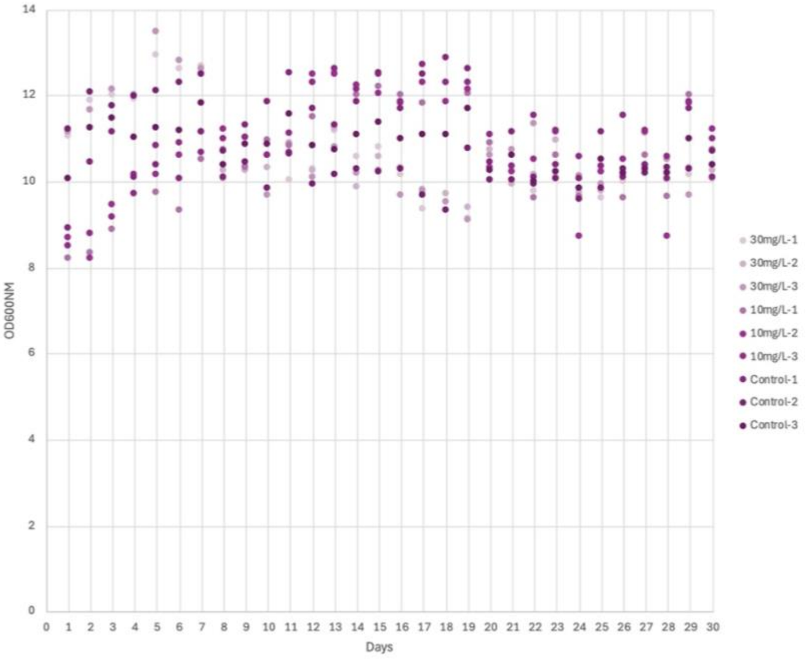

Figure S2: Cellular growth of yeast strains (not)-exposed to Deltamethrin at the end of each day during 30-day exposure. 10 mg/L, 30 mg/L and control strains after dash indicates the number of replicates.
